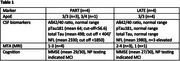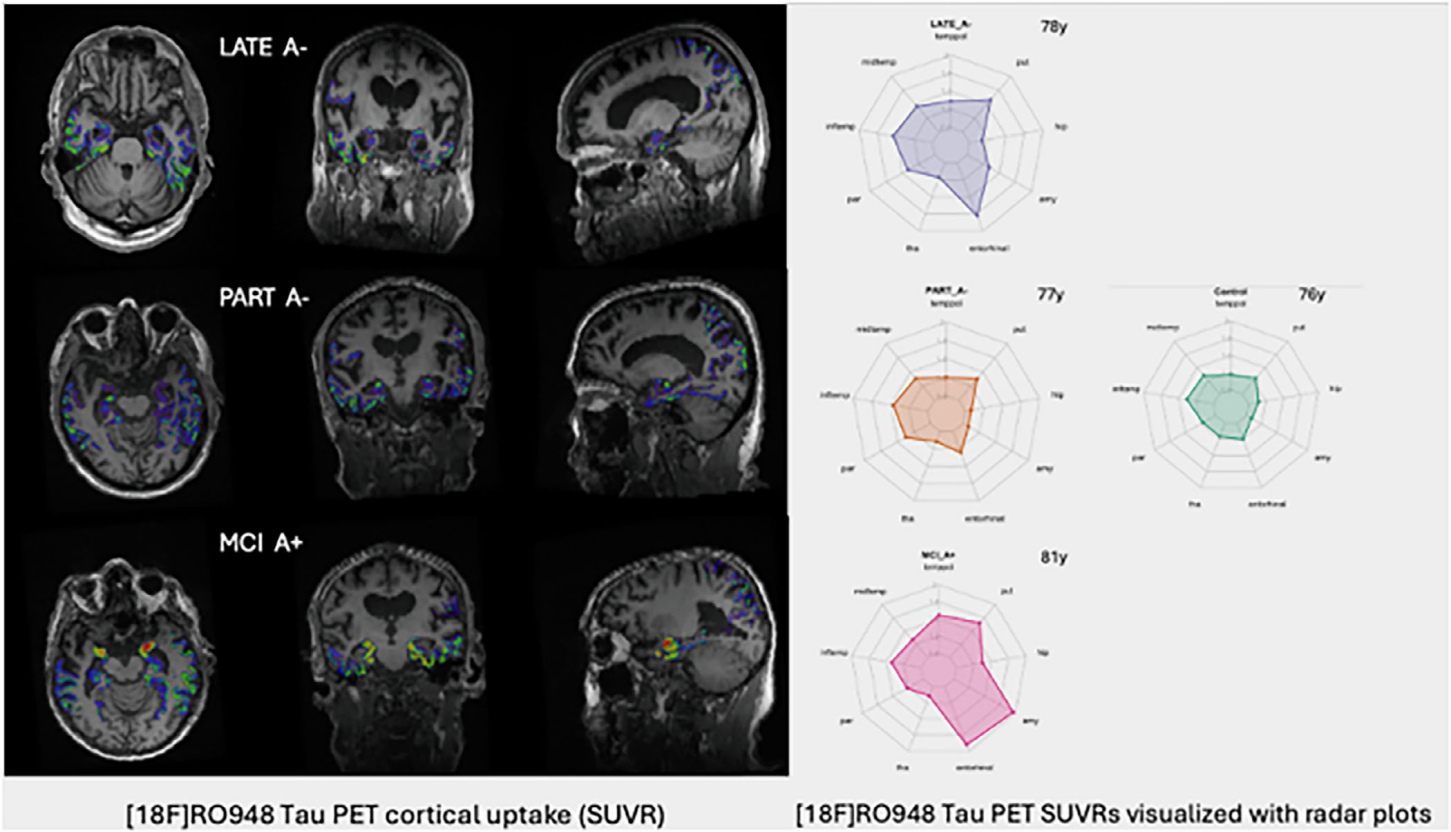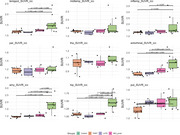# [18F] RO948 Tau PET imaging and plasma biomarkers in PART and LATE patients compared with sporadic Alzheimer's Disease

**DOI:** 10.1002/alz70856_107395

**Published:** 2026-01-11

**Authors:** Agneta K Nordberg, Marco Bucci, Mariola Zapater‐Fajari, Konstantinos Chiotis, Anders Wall, Jonas Eriksson, Gunnar Antoni, Ilaria Pola, Kübra Tan, Wiebke Traichel, Andrea L. Benedet, Nicholas Ashton, Kaj Blennow, Henrik Zetterberg, Nenad Bogdanovic

**Affiliations:** ^1^ Theme Inflammation and Aging, Karolinska University Hospital, Stockholm, Sweden; ^2^ Department of Neurobiology, Care Sciences and Society, Division of Clinical Geriatrics, Center for Alzheimer Research, Karolinska Institutet, Stockholm, Sweden; ^3^ Division of Clinical Geriatrics, Center for Alzheimer Research, Department of Neurobiology, Care Sciences and Society, Karolinska Institutet, Stockholm, Sweden; ^4^ Turku PET Centre, Turku University Hospital, University of Turku and Åbo Akademi University, Turku, Finland; ^5^ Department of Neurobiology, Care Sciences and Society, Center for Alzheimer Research, Division of Clinical Geriatrics, Karolinska Institutet, Stockholm, Sweden; ^6^ Department of Neurology, Karolinska University Hospital, Stockholm, Sweden; ^7^ Department of Surgical Sciences, Section of Nuclear Medicine & PET, Uppsala University, Uppsala, Sweden; ^8^ Department of Medicinal Chemistry, Uppsala University, Uppsala, Sweden; ^9^ PET Centre, Uppsala University Hospital, Uppsala, Sweden; ^10^ Department of Psychiatry and Neurochemistry, Institute of Neuroscience and Physiology, The Sahlgrenska Academy, University of Gothenburg, Mölndal, Sweden; ^11^ King's College London, Institute of Psychiatry, Psychology & Neuroscience, Maurice Wohl Clinical Neuroscience Institute, London, United Kingdom; ^12^ Clinical Neurochemistry Laboratory, Sahlgrenska University Hospital, Mölndal, Sweden; ^13^ Clinical Neurochemistry Laboratory, Sahlgrenska University Hospital, Mölndal, Västra Götalands län, Sweden; ^14^ Wisconsin Alzheimer's Disease Research Center, University of Wisconsin‐Madison, School of Medicine and Public Health, Madison, WI, USA; ^15^ UCL Queen Square Institute of Neurology, London, United Kingdom; ^16^ Hong Kong Center for Neurodegenerative Diseases, Hong Kong, Hong Kong, China; ^17^ Department of Neurobiology, Care Sciences and Society, Division of Clinical Geriatrics, Center for Alzheimer Research, Karolinska University, Stockholm, Sweden

## Abstract

**Background:**

Although the diagnosis of Alzheimer´s disease (AD) dominates in the tertiary memory clinic setting, there are also patients which show no sign for presence of amyloid in brain when assessed for CSF biomarkers after lumbar puncture (LP) or amyloid PET. Since these amyloid negative (A‐) patients can clinically mimic symptomatic AD patients, it is important to obtain further insight into the in vivo pathology of these patients. This study therefore aimed to perform tau PET imaging with the tracer [18F]RO948 and measure plasma biomarkers in patients clinically diagnosed as primary age‐related tauopathy (PART) and limbic dominant TDP‐43 age‐related encephalopathy (LATE) at the clinic for cognitive disorders at Karolinska University Hospital.

**Method:**

Four patients diagnosed with **PART** (mean age 76 years) and four with **LATE** (mean age 79 years) were included in the study. Clinical characteristics and biomarkers are reported in Table 1. The ATN classification for PART patients was A‐T+N+ and for LATE patients A‐T‐N+. On the same day, all participants underwent [18F]RO948 tau PET and MRI scans, and blood sampling for plasma biomarker analysis using the NuLISAseq (Alamarbio) CNS panel. The obtained data was compared with 27 amyloid positive MCI and AD patients from Karolinska as well as 10 cognitive healthy controls.

**Result:**

Low uptake of [18F]RO948 was observed in PART and LATE brains compared to MCI A+ as shown in Figure 1, including also Radar plots of different brain regions. Box plot data (Figure 2) showed low [18F]RO948 regional uptake except for a higher uptake (*p* <0.05) in the putamen in PART and LATE compared to controls. Higher plasma levels of ptau217, ptau181, ptau231 were observed in PART patients (*p* <0.05) but not in LATE compared to cognitively healthy controls. Plasma ptau217 levels were however higher in LOAD (*p* <0.05) compared to PART. Higher plasma Aß42 values were observed both in LATE and PART compared to LOAD.

**Conclusion:**

PART and LATE patients exhibit Tau PET uptake similar to that of controls, except in the putamen. Additionally, PART patients show elevated plasma levels of *p*‐tau 217, *p*‐tau 181, and *p*‐tau 231, whereas LATE patients do not.